# Adult cor-triatriatum sinistrum—a sinistral lesion with varied presentations: a case series

**DOI:** 10.1093/ehjcr/ytag114

**Published:** 2026-06-22

**Authors:** Apratim Roy Choudhury, Jineesh Valakkada, Anoop Ayyappan, Ajay Alex, Venkata Subbaih Arunachalam, Smily Sharma

**Affiliations:** Department of Imaging Sciences and Interventional Radiology, Sree Chitra Tirunal Institute for Medical Sciences and Technology (SCTIMST), Medical College P.O., Trivandrum 695011, Kerala, India; Department of Imaging Sciences and Interventional Radiology, Sree Chitra Tirunal Institute for Medical Sciences and Technology (SCTIMST), Medical College P.O., Trivandrum 695011, Kerala, India; Department of Imaging Sciences and Interventional Radiology, Sree Chitra Tirunal Institute for Medical Sciences and Technology (SCTIMST), Medical College P.O., Trivandrum 695011, Kerala, India; Department of Imaging Sciences and Interventional Radiology, Sree Chitra Tirunal Institute for Medical Sciences and Technology (SCTIMST), Medical College P.O., Trivandrum 695011, Kerala, India; Department of Imaging Sciences and Interventional Radiology, Sree Chitra Tirunal Institute for Medical Sciences and Technology (SCTIMST), Medical College P.O., Trivandrum 695011, Kerala, India; Department of Imaging Sciences and Interventional Radiology, Sree Chitra Tirunal Institute for Medical Sciences and Technology (SCTIMST), Medical College P.O., Trivandrum 695011, Kerala, India

**Keywords:** Case series, Congenital heart disease, CT, MR, Cardiac imaging, Cor triatriatum, Cor triatriatum sininstrum, Levo-atrio-cardinal vein

## Abstract

**Background:**

Cor-triatriatum sinistrum (CTS) is a rare congenital heart disease, characterized by a membrane dividing the left atrium into two chambers. Echocardiography is the primary imaging modality. However, computed tomography and magnetic resonance imaging both play important roles in delineating the anatomy of the condition and also determining the associated anomalies. Although thought to be a disease of childhood, CTS can also present in adulthood with varied clinical manifestations like dyspnea, palpitations, and syncopal attacks. In CTS, the left atrial membrane causes pulmonary venous obstruction and eventual pulmonary arterial hypertension. It is usually associated with membrane fenestrations, which allow some antegrade flow. If not, there are relief valves, such as the ostium secundum atrial septal defect (OS-ASD) and/or the levo-atrial-cardinal vein.

**Case summary:**

We present three patients with CTS. The first patient is of Type I CTS—presenting with CTS and an OS-ASD, which is acting as a relief valve. The second patient is of Type II CTS with associated levo-atrio-cardinal vein and venovenous shunting. The third patient presented with CTS with partial anomalous pulmonary venous connection (PAPVC)—suggestive of Type III CTS. All the cases were managed surgically with good outcomes.

**Discussion:**

CTS, though thought to be a disease of childhood, may present in adults with varied clinical presentations. Imaging plays a crucial role in the diagnosis and delineation of associated anomalies. Management is usually surgical with a good clinical outcome.

Learning points

- Cor-triatriatum sinistrum (CTS), although considered a childhood condition, can also occur in adulthood with diverse clinical presentations.
- In CTS, the left atrial membrane causes pulmonary venous obstruction and eventual pulmonary arterial hypertension.
- Multimodality imaging plays a crucial role in detecting the membrane and also the associated anomalies.

## Introduction

Cor-triatriatum, meaning three-chambered atria, is a rare congenital heart disease.^1^ It is a type of pulmonary venous abnormality.^2^ Cor-triatriatum sinistrum (CTS) is characterized by a membrane that divides the left atrium (LA) into a proximal pulmonary venous chamber (PVC), which receives the pulmonary venous blood and a distal left atrial chamber (LAC) with the mitral annulus and the left atrial appendage.^1^ CTS usually presents in infancy or childhood with pulmonary venous hypertension.^3^ Adult CTS is quite rare, with echocardiography and cross-sectional imaging playing crucial roles in diagnosis.^4^ In this case series, we describe the clinical and imaging features of three cases of CTS presenting in adulthood.

## Summary figure

**Figure:**
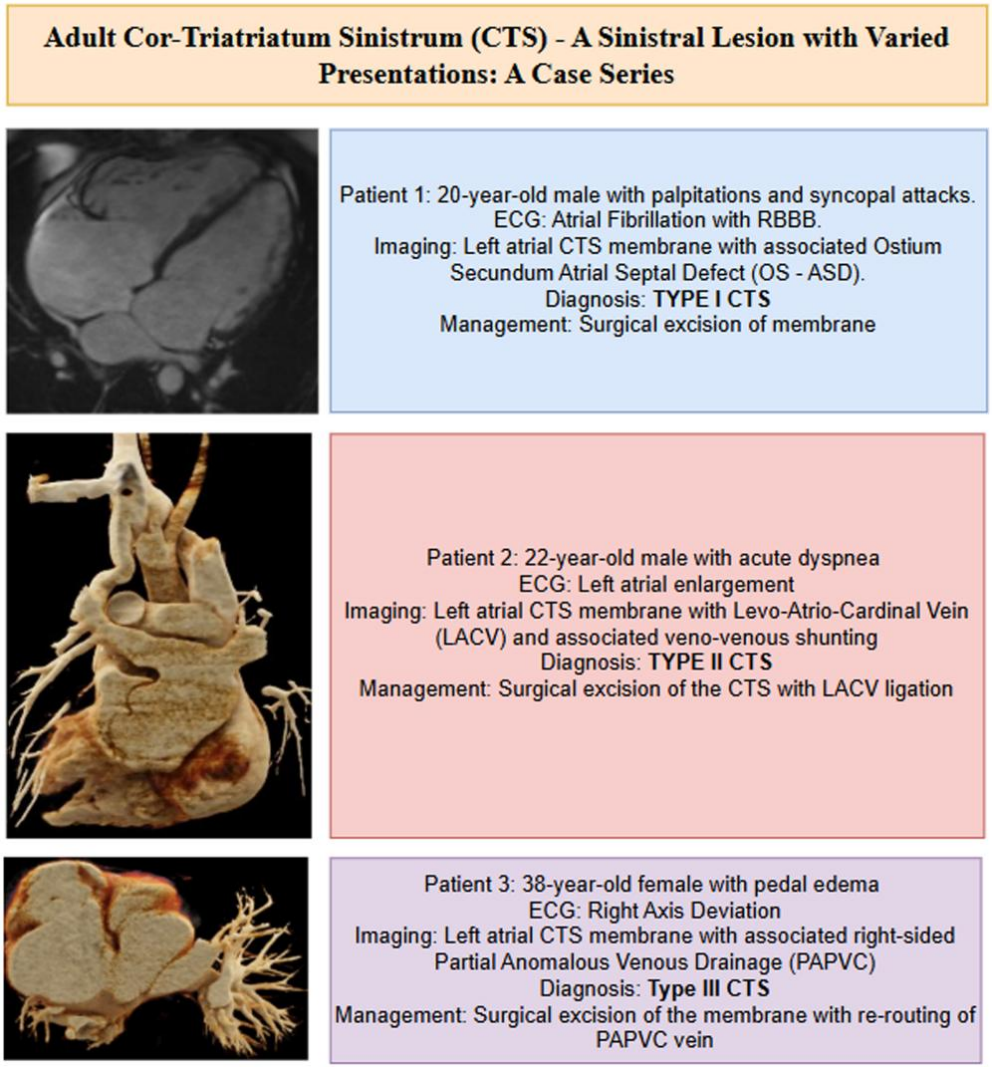


### Patient 1

**Figure 1:**
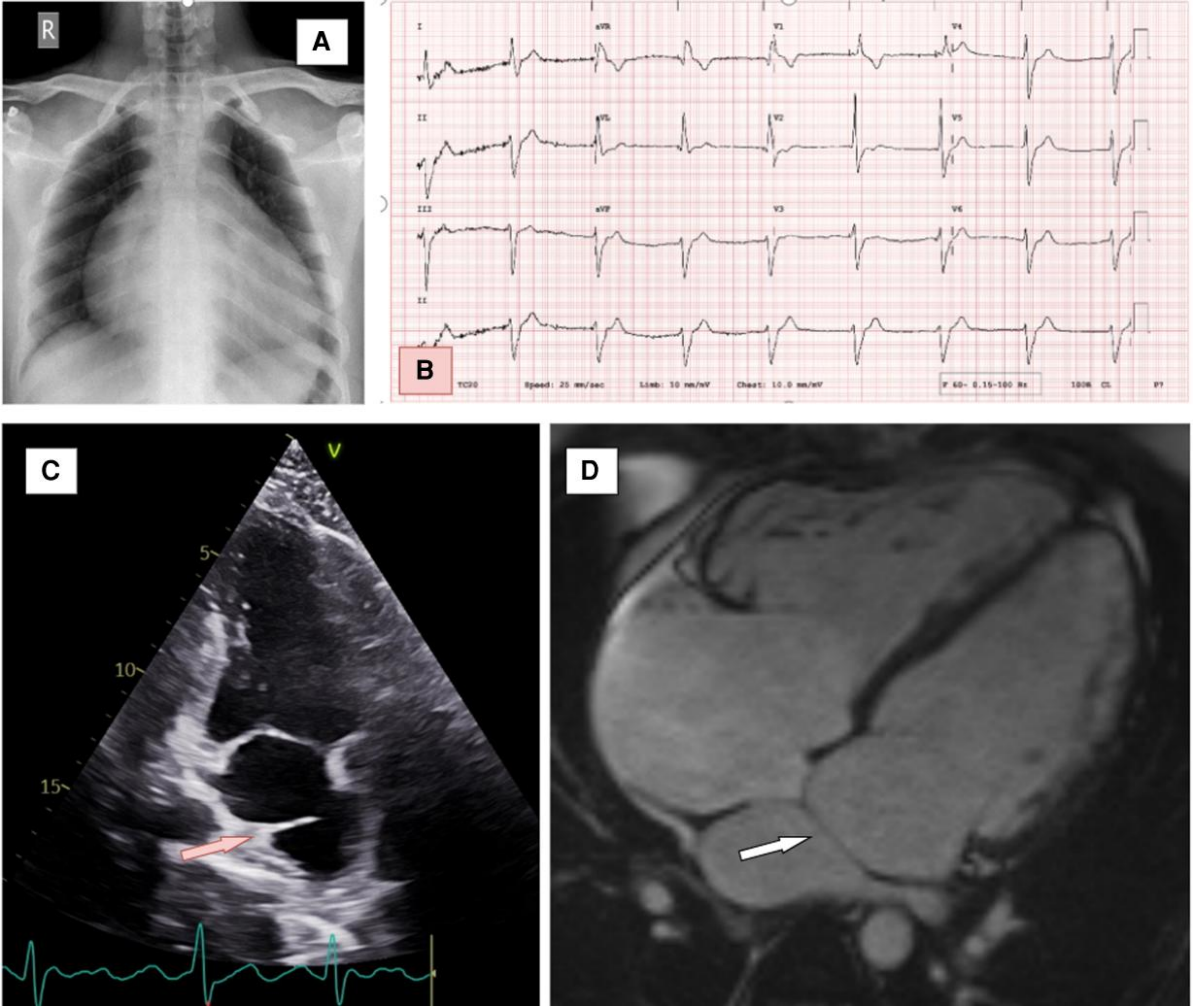
A 20-year-old male patient presented with recurrent episodes of palpitations and syncopal attacks. The chest X-ray postero-anterior view (A) shows cardiomegaly with right atrial enlargement. Electrocardiogram (B) shows atrial fibrillation with junctional rhythm and extreme right axis deviation. The echocardiogram (C) shows a thin membrane (red arrow) in the left atrium, dividing the chamber into two parts. The magnetic resonance imaging 4-chamber view (D) shows the cor-triatriatum membrane in the left atrium (white arrow). The right atrium and the right ventricle are dilated in the magnetic resonance imaging due to left-to-right shunt via an ostium secundum atrial septal defect (not shown). The patient underwent excision of the cor-triatriatum membrane, atrial septal defect patch closure with left bundle branch pacing.

A 20-year-old male patient presented with recurrent episodes of palpitations and syncopal attacks over the past six months. Each episode lasted for 5–6 min and was not associated with convulsions or palpitations. On physical examination, he was comfortable at rest, had a pulse rate of 48 beats per minute (bpm), blood pressure of 120/70 and an SpO_2_ of 98% on room air. There was no evidence of any pallor, cyanosis, jaundice, or prominent jugular waves. Both the heart sounds were well audible. A chest radiograph (*Figure 1A*) revealed cardiomegaly, and an electrocardiogram (ECG) (*Figure 1B*) showed junctional rhythm, atrial fibrillation with a right bundle branch block, a wide QRS complex, and extreme right axis deviation. An echocardiogram (*Figure 1C*) showed a thin membrane in the LA, along with an ostium secundum atrial septal defect (OS-ASD) and a left-to-right shunt. Cardiac magnetic resonance imaging (MRI) (*Figure 1D*) demonstrated the presence of the CTS membrane, which divided the LA into a postero-superior PVC and an antero-inferior LAC. The associated OS-ASD resulted in left-to-right shunting, leading to dilatation of the right-sided cardiac chambers. The patient underwent surgical excision of the membrane, which contained multiple fenestrations. Following the surgery, the patient continued to have junctional rhythm with a widened QRS complex and hence was paced with left bundle branch pacing and was discharged on amiodarone.

### Patient 2

**Figure 2:**
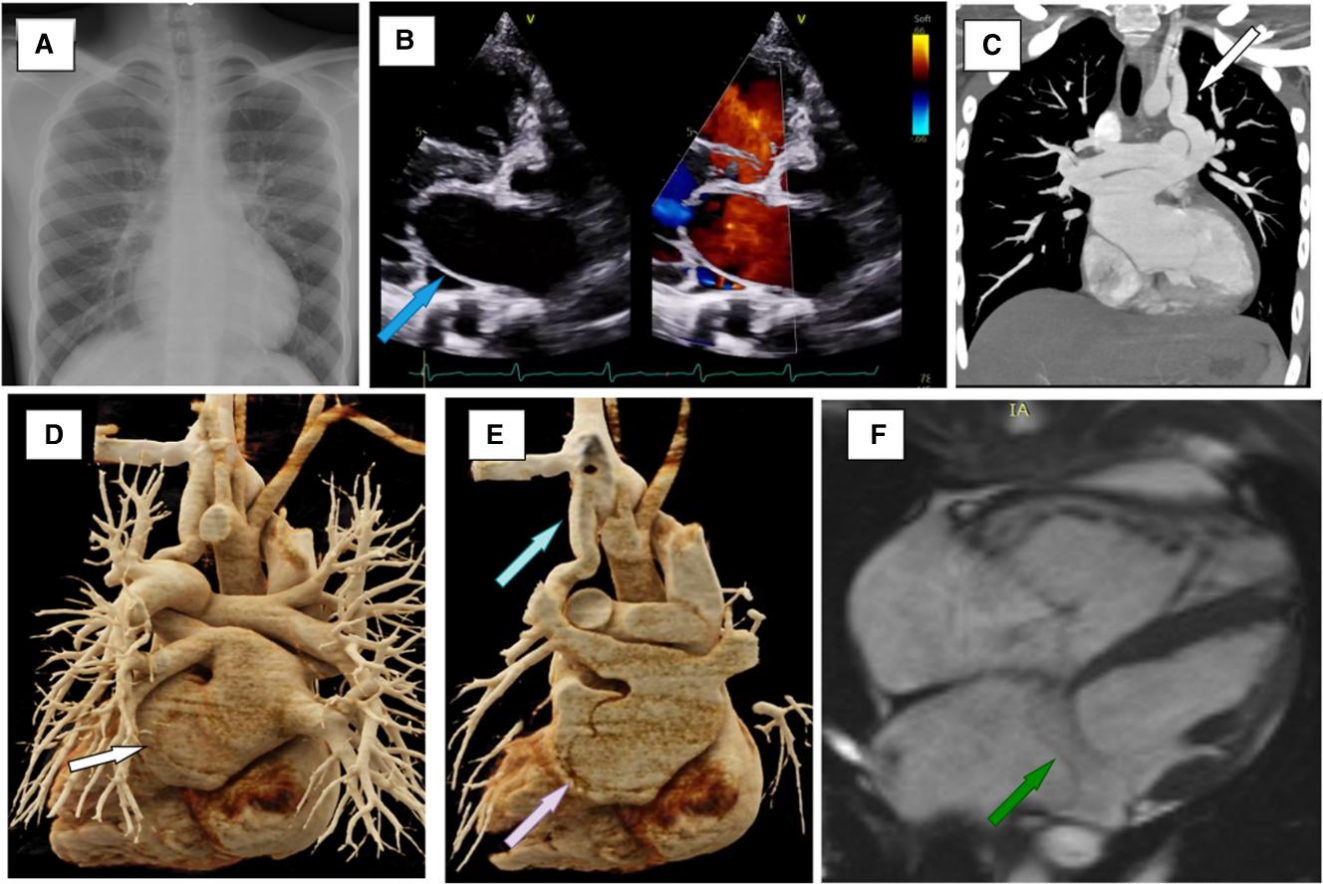
A 22-year-old male patient presented with an acute onset of breathlessness. Chest X-ray postero-anterior (A) view shows mild Grade I pulmonary venous congestion with cephalization in the upper lobe of the lung. Echocardiogram (*B*) shows a dilated left atrium with a thin membrane (blue arrow) dividing the left atrial cavity into two chambers—suggestive of cor-triatriatum. Computed tomography angiogram coronal view (C) demonstrates the levo-atrio-cardinal vein (white arrow) connecting the left upper lobe pulmonary vein to the left innominate vein. The cinematic virtual reconstruction technique images show (D and E) the thin left atrial membrane (white arrow in D and violet arrow in E) along with the levo-atrio-cardinal vein (blue arrow in E). The magnetic resonance imaging 4-chamber view (F) demonstrates the left atrial membrane (green arrow). The patient underwent excision of the membrane with ligation of the levo-atrio-cardinal vein.

A 22-year-old male patient presented with an acute onset of breathlessness. His vital signs indicated a pulse of 110 bpm, blood pressure of 100/70 mmHg, and oxygen saturation of 90% in room air. He was conscious, oriented, and comfortable at rest with no pallor, cyanosis, jaundice, or pedal edema. ECG revealed signs of left atrial enlargement, while a chest X-ray (CXR) (*Figure 2A*) showed interstitial edema and pulmonary venous congestion. The echocardiogram (*Figure 2B*) identified a thin membranous structure within the LA, which divided the left atrial cavity into two chambers, although no shunt was observed. Additionally, pulmonary artery pressure was found to be elevated. Cardiac computed tomography (CT) (*Figure 2C, 2D,* and *2E*) demonstrated a levo-atrio-cardinal vein (LACV) that was shunting blood from the left superior pulmonary vein to the left innominate vein. Notably, the left-sided cardiac chambers appeared hypoplastic in comparison to the right atrium (RA) and right ventricle (*Figure 2F*), with dilated pulmonary veins and arteries. The patient subsequently underwent surgical resection of the membrane and ligation of the LACV, resulting in significant clinical improvement. In this case, fenestrations were absent in the CTS membrane.

### Patient 3

**Figure 3:**
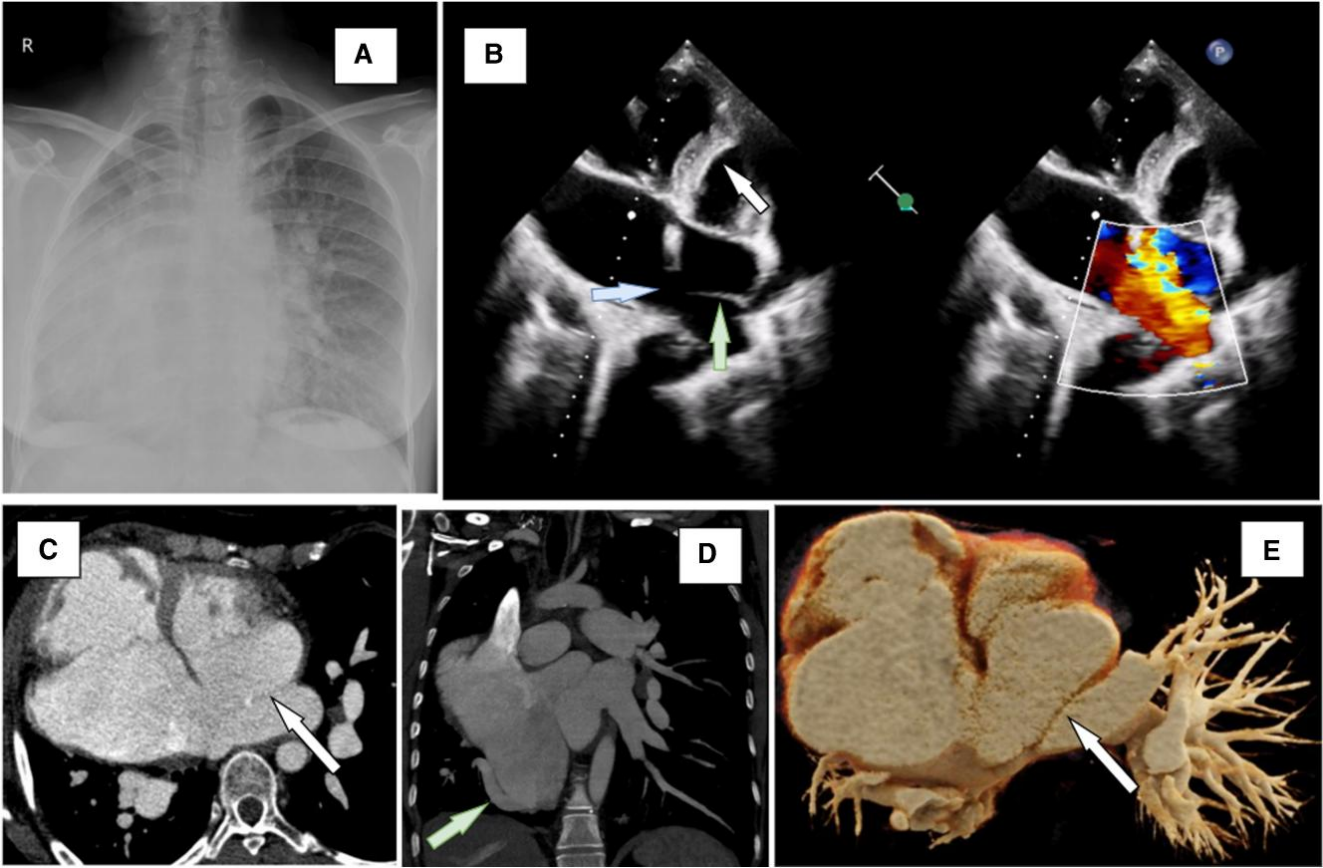
A 38-year-old woman presented with bilateral pedal edema. The chest X-ray postero-anterior view (A) shows cardiomegaly with the cardiac shadow occupying the majority of the right hemithorax. The 4-chamber view of the echocardiogram (B) shows a thin membrane in the left atrial cavity (green arrow) along with a large ostium secundum atrial septal defect (blue arrow). The cardiac computed tomography images (C and D) demonstrate mesocardia, left atrial membrane, a large atrial septal defect, and a dilated right atrial cavity due to left-to-right shunt via the atrial septal defect. Note is also made of anomalous drainage of the right lower lobe pulmonary vein near the right atrium-inferior vena cava junction (green arrow in D). Cinematic virtual reconstruction technique image (E) shows the cor-triatriatum membrane (white arrow).

A 38-year-old woman presented with gradually progressive edema in her feet. Clinical examination revealed tender hepatomegaly. CXR (*Figure 3A*) showed cardiomegaly with dextroposition of the heart. ECG showed extreme right axis deviation with right ventricular hypertrophy. The echocardiogram (*Figure 3B*) revealed a thin membrane in the LA with ostium secundum ASD, a massively dilated right atrium, and dilated pulmonary arteries. Cardiac CT revealed a thin membrane in the LA, dividing it into two chambers. The proximal PVC received drainage from the left lung's veins. However, the right-sided pulmonary veins formed an anomalous connection, draining into the right atrium-inferior vena cava junction (*Figure 3C, 3D,* and *3E*). Additionally, there was a large ASD with a left-to-right shunt. Due to reduced blood flow, the left ventricle was small and hypoplastic. In contrast, the right-sided chambers and the pulmonary artery were dilated. The patient underwent surgical repair involving membrane excision in the LA, rerouting of the right-sided PAPVC, and closure of the ASD. A small 5 mm patent foramen ovale was left behind as the relief.

## Discussion

CTS is a rare congenital heart defect with an incidence of 0.4%.^5^ This condition is characterized by a fibro-muscular septum that divides the LA into two separate chambers. One chamber, located in the postero-superior position, receives pulmonary venous drainage, while the second chamber, found in the antero-inferior position, contains the mitral valve and the left atrial appendage (LAA).^6^

The embryological basis of CTS can be explained through three primary theories: mal-septation, mal-incorporation, and entrapment.^7^ The mal-septation theory suggests that the CTS membrane arises from the abnormal development of the septum primum. The mal-incorporation theory, which is the most widely accepted among scholars, posits that CTS results from the inadequate fusion of the common pulmonary vein chamber with the primitive LA. The entrapment theory implies that the left horn of the sinus venosus traps the developing common pulmonary vein, hindering its incorporation into the primitive LA.

Traditionally, CTS is classified into three distinct types.^1^ In Type I and Type II, the PVC receives drainage from all four pulmonary veins; however, Type I features communication with the LA through a fenestration, while Type II lacks both fenestration and LA communication. Consequently, Type II CTS typically exhibits a relief valve outflow, such as an atrial septal defect (ASD) or a venous collateral. Our first case involved Type I CTS, characterized by small fenestrations within the CTS membrane. The second case represented Type II CTS, with no fenestrations, where the left atrial common vein (LACV) functioned as a relief valve to the PVC. Type III CTS is categorized as subtotal CTS, as observed in our third case, where the pulmonary veins from one lung drain into the PVC, while the other lung demonstrates anomalous drainage into the systemic or portal vein.

CTS is predominantly recognized as a pediatric condition; however, adult cases have also been documented in the literature. The degree of obstruction imposed by the membrane significantly influences the onset of clinical symptoms.^4^ In pediatric patients, common manifestations include pulmonary edema, respiratory distress, and heart failure. In instances of pulmonary hypertension due to pulmonary venous congestion, right-to-left shunting may occur from the higher-pressure RA to the lower-pressure LA, potentially leading to cyanosis via an ostium secundum ASD (OS-ASD) or a patent foramen ovale.^7^

In adult patients, symptoms can vary significantly and may include cough, dyspnea on exertion, pulmonary edema, paroxysmal nocturnal dyspnea, syncope, atrial fibrillation, and the formation of atrial clots. The high-velocity blood flow through the thin membrane of the CTS may cause damage to the mitral leaflets, resulting in secondary mitral regurgitation (MR). In such cases, the MR jet remains confined within the LA, while the CTS membrane bulges downward, giving rise to the so-called ‘helmet sign.’^8^ Our first two cases demonstrated mild secondary MR due to damage to the mitral leaflets.

Imaging constitutes a cornerstone of diagnosis, with echocardiography, cardiac CT, and cardiac MRI serving essential complementary roles. The primary objectives of imaging include the visualization of the fibro-muscular membrane, the identification of additional anomalies such as ASD or anomalous pulmonary venous drainage, and the assessment of pulmonary hypertension. MRI can quantitatively evaluate the shunt fraction and assess ventricular function.^9^

The three cases discussed in this series exemplify the diverse presentations, physiological characteristics, and imaging findings associated with adult CTS. The first case presented with syncopal episodes, likely attributable to pulmonary arterial hypertension (PAH) and reduced ventricular ejection fraction. In this instance, the fenestrations in the CTS served as a relief valve to the dilated LA. The second patient exhibited atrial fibrillation due to significant atrial dilation, with the associated OS-ASD functioning as a relief valve to alleviate pressure in the LA, in conjunction with small fenestrations in the CTS membrane. Notably, this case lacked fenestrations and associated ASD; instead, a rare venous anomaly, the LACV, served as the relief valve, indicating elevated LA pressures. The third patient presented with symptoms consistent with right heart failure, and imaging revealed both ASD and pulmonary arterial persistent venous connections (PAPVC), contributing to left-to-right shunting and the development of PAH.

Differential diagnosis of CTS from a supramitral membrane via imaging is essential.^10^ Key distinctions have been summarized in the *Table 1*. Other differential diagnoses may include congenital or acquired mitral stenosis (MS).^11^ Imaging effectively differentiates between MS and CTS, as MS is categorized as a valvular pathology, while CTS represents a supra-valvar pathology. The management of CTS is predominantly surgical.^11^ Symptomatic CTS cases presenting in adulthood should undergo surgical intervention, and post-repair life expectancy for patients with CTS may ultimately align with that of the general population, provided there are no additional associated cardiac defects.

**Table 1:** Summarizing the difference between the cor-triatriatum and the supra-valvar stenosis ring

Cor triatriatum	Supra-valvar stenosing ring
Mobile during diastole due to windsock effect	Immobile; adherent to base of mitral valve
Curvilinear membrane	Circumferential
Foramen ovale lies distal to membrane	Foramen ovale lies proximal to membrane
Left atrial appendage located distal to membrane	Left atrial appendage located proximal to the membrane

## Conclusion

These cases illustrate the wide range of clinical presentations in adults with CTS. Imaging is crucial for accurate diagnosis, as it helps to delineate the membrane and identify other potential abnormalities, such as atypical venous drainage, associated mitral regurgitation, and pulmonary hypertension, while also assessing left ventricular function. Management of CTS predominantly involves surgical intervention, which typically results in favorable postoperative outcomes.

## Data Availability

The data underlying this article will be shared on reasonable request to the corresponding author.

## Lead author biography

Dr. Apratim Roy Choudhury is a Cardiovascular and Interventional Radiology resident from Sree Chitra Tirunal Institute for Medical Sciences and Technology (SCTIMST), Kerala, India. He has led the current article under the supervision of Dr. Smily Sharma (Assistant Professor) and Dr. Jineesh Valakkada (Additional Professor) and the Cardiovascular and Interventional Radiology team from SCTIMST.

## Notes

**Consent:** The authors confirm that written consent for submission and publication of this case, including images and associated text, has been obtained from the patient in line with COPE guidance.
